# Bell on Baths, the Watery Regimen, Hydropathy, Pulmonary Inhalation, &c.

**Published:** 1850-09

**Authors:** 


### ARTICLE II.

*A Treatise on Baths; including Cold, Sea, Warm, Hot, Vapor, Gas, and Mud Baths: also, on the Watery Regimen, Hydropathy, and Pulmonary Inhalation; with a Description of Bathing in Ancient and Modern Times.*—By JOHN BELL, M. D., Member of the American Medical Association, and of the Pennsylvania Medical Society; Fellow of the College of Physicians of Philadelphia; Member of the American Philosophical Society; formerly lecturer on the Institutes of Medicine, and Materia Medica. Philadelphia: Barrington & Haswell, 1850; pp., 658; 12mo. (From the Publishers.) For sale by J. Keen, Jr., & Brother.

This is decidedly the most readable medical book of the year, and though not written exclusively for the perusal of the profession, it is one from which the physician will derive much solid and valuable information, as well as a great fund of entertainment. The learned and accomplished author has, from a vast mass of heterogenous and ill-assorted literature on the subject of which he treats, elicited order out of chaos, and presented us with a volume at once learned, elegant, and in-instructive.

The delusions of Hydropathy, and the real value of water as a therapeutic agent, are fairly and judiciously set forth.—The importance of bathing is eloquently urged, and the directions for the particular kind of bath required under given circumstances are plain and perspicuous. To the classical scholar, the description of the ancient baths possesses an interest, as leading him again over ground hallowed by scholastic associations. And interspersed through the whole, is a fund of quaint anecdote, and curious learning, which cannot fail to endear the volume and the author to every one who has in him the least spice of the humorist or antiquary. In short, any one who reads Prof. Bell's book, without being interested with it, may, like Dogberry, insist with great propriety on having it written down that he is an ass. M.

